# Safety and pharmacokinetics of MM-302, a HER2-targeted antibody–liposomal doxorubicin conjugate, in patients with advanced HER2-positive breast cancer: a phase 1 dose-escalation study

**DOI:** 10.1038/s41416-018-0235-2

**Published:** 2018-10-26

**Authors:** Pamela Munster, Ian E. Krop, Patricia LoRusso, Cynthia Ma, Barry A. Siegel, Anthony F. Shields, István Molnár, Thomas J. Wickham, Joseph Reynolds, Karen Campbell, Bart S. Hendriks, Bambang S. Adiwijaya, Elena Geretti, Victor Moyo, Kathy D. Miller

**Affiliations:** 1 Helen Diller Family Comprehensive Cancer Center, Department of Medicine, University of California, San Francisco, CA USA; 2 Department of Medical Oncology, Dana-Farber Cancer Institute, Boston, MA USA; 3 Yale Cancer Center, New Haven, CT USA; 4 Department of Medicine and the Alvin J. Siteman Cancer Center, Washington University School of Medicine, St. Louis, MO USA; 5 Mallinckrodt Institute of Radiology and the Alvin J. Siteman Cancer Center, Washington University School of Medicine, St. Louis, MO USA; 6 Department of Oncology, Karmanos Cancer Institute, Wayne State University, Detroit, MI USA; 7 Research and Development, Merrimack Pharmaceuticals, Inc, Cambridge, MA USA; 8 Indiana University Melvin and Bren Simon Cancer Center, Indianapolis, IN USA

**Keywords:** Breast cancer, Phase I trials

## Abstract

**Background:**

This phase 1 dose-escalation trial studied MM-302, a novel HER2-targeted PEGylated antibody–liposomal doxorubicin conjugate, in HER2-positive locally advanced/metastatic breast cancer.

**Methods:**

Patients were enrolled in four cohorts: MM-302 monotherapy (8, 16, 30, 40, and 50 mg/m^2^ every 4 weeks [q4w]); MM-302 (30 or 40 mg/m^2^ q4w) plus trastuzumab (4 mg/kg q2w); MM-302 (30 mg/m^2^) plus trastuzumab (6 mg/kg) q3w; MM-302 (30 mg/m^2^) plus trastuzumab (6 mg/kg) and cyclophosphamide (450 mg/m^2^) q3w.

**Results:**

Sixty-nine patients were treated. The most common adverse events (AEs) were fatigue and nausea. Grade 3/4 AEs of special interest included neutropenia, fatigue, mucosal inflammation, anemia, thrombocytopenia, febrile neutropenia, and palmar-plantar erythrodysesthesia. The MTD was not reached. With MM-302 ≥ 30 mg/m^2^, overall response rate (ORR) was 13% and median progression-free survival (mPFS) 7.4 months (95% CI: 3·5–10·9) in all arms. In 25 anthracycline-naïve patients, ORR was 28·0% and mPFS 10·9 months (95% CI: 1·8–15·3). Imaging with ^64^Cu-labeled MM-302 visualized tumor-drug penetrance in tumors throughout the body, including the brain.

**Conclusion:**

MM-302 monotherapy, in combination with trastuzumab, or trastuzumab plus cyclophosphamide, was well tolerated and showed promising efficacy. The selected phase 2 MM-302 dose was 30 mg/m^2^ plus 6 mg/kg trastuzumab q3w.

## Background

Human epidermal growth factor receptor 2 (HER2)-targeted anticancer therapies such as trastuzumab, pertuzumab, and ado­trastuzumab emtansine (T-DM1) have transformed the treatment of HER2-positive advanced/metastatic breast cancer.^1–3^ Despite these improvements, the disease ultimately progresses, and new treatment strategies are required.

Anthracyclines, such as doxorubicin, are efficacious in the treatment of HER2-positive breast cancer.^4–6^ However, their use in early-stage trastuzumab-containing breast cancer regimens is limited by their adverse effects on the heart,^2^ and some studies show that a taxane-based regimen may have similar efficacy in the adjuvant and neoadjuvant settings.^6,7^ There is a strong need for efficacious regimens after taxane failure in the metastatic setting, and less toxic early-stage regimens. Compared with free doxorubicin, liposomal formulations of doxorubicin have less cardiac toxicity and fewer side effects.^8–16^ The introduction of targeted antibody conjugates has provided great promise to maximize cancer cell uptake while minimizing systemic toxicity. Targeted delivery of doxorubicin to HER2-overexpressing cells would allow use of one of the most effective chemotherapeutic agents while sparing the heart.

MM-302 is a HER2-targeted PEGylated antibody–liposomal doxorubicin conjugate that targets HER2­overexpressing tumor cells, delivers doxorubicin to tumor cells, and limits exposure to healthy cells such as cardiomyocytes (Supplemental Fig. 1).^17,18^ In preclinical models of HER2-positive breast cancer, MM-302 demonstrated superior antitumor activity compared to both doxorubicin and PEGylated liposomal doxorubicin.^18^ MM­302 and trastuzumab bind different epitopes of HER2, and the combination of MM-302 and trastuzumab demonstrated enhanced antitumor activity compared with either agent alone in HER2­overexpressing tumor xenograft models.^12,19–21^ Preclinical studies have also shown that cyclophosphamide pretreatment increased the delivery and antitumor activity of MM-302.^22^ These findings support clinical investigation of MM-302 in combination with trastuzumab and trastuzumab plus cyclophosphamide. Here we report the results from the first-in-human phase 1 study of MM-302 in patients with advanced HER2-positive breast cancer.

## Methods

### Study design

This phase 1, open-label, multicenter, dose-escalation study evaluated the safety, tolerability, and pharmacokinetics (PK) of MM-302 as a monotherapy (arm 1), in combination with trastuzumab (arm 2 and arm 3), or trastuzumab plus cyclophosphamide (arm 4) in patients with advanced HER2-positive breast cancer (ClinicalTrials.gov Identifier: NCT01304797).

The primary objectives were to assess safety and tolerability, and to determine the maximum tolerated dose (MTD) and phase 2 dose of MM-302 as a monotherapy, in combination with trastuzumab or trastuzumab plus cyclophosphamide. Secondary objectives were to describe dose-limiting toxicities (DLTs), objective response rate (ORR; confirmed complete response [CR] or partial response [PR]), clinical benefit rate (CBR; confirmed CR, PR or stable disease at 24 weeks), duration of response, progression-free survival (PFS), immunogenicity and PK of MM-302 in patients enrolled in arm 1–4. Exploratory objectives investigated the correlation of clinical activity with levels of HER2 amplification or protein expression, and MM-302 biodistribution and tumor deposition.

The trial was conducted at five sites in the United States and was approved by the Institutional Review Board at each site. All patients provided written informed consent prior to study enrollment in accordance with the Declaration of Helsinki, the Code of Federal Regulations and local regulatory requirements.

### Patients

Key inclusion criteria were: histologically or cytologically confirmed HER2-positive locally advanced/metastatic breast cancer; tumor amenable to biopsy; measurable disease per Response Evaluation Criteria in Solid Tumors (RECIST) version 1·1; age ≥ 18 years; Eastern Cooperative Oncology Group (ECOG) performance status 0 or 1; left ventricular ejection fraction (LVEF) ≥ 50%; adequate renal/hepatic function and bone marrow reserves. Patients with stable, treated central nervous system metastases were allowed.

Key exclusion criteria were: previous cumulative anthracycline total dose of > 300 mg/m^2^ doxorubicin equivalent; significant cardiovascular problems.

### Treatment

MM-302 (Merrimack Pharmaceuticals, Cambridge, MA, USA) was administered by intravenous infusion over 60 min and the dose based on a doxorubicin hydrochloride equivalent basis. Patients were sequentially enrolled in one of four arms (Table 1). A standard ‘3 + 3’ dose escalation design was used starting with MM-302 monotherapy (arm 1; 8, 16, 30, 40, and 50 mg/m^2^) administered once every 4 weeks (q4w) with expansion cohorts at 40 and 50 mg/m^2^ (each containing 12 patients, respectively). Arm 2 was a combination dose escalation of intravenous MM-302 (30 and 40 mg/m^2^ q4w) plus intravenous trastuzumab (4 mg/kg with 6 mg/kg loading) every 2 weeks (q2w). Arm 3 cohort 1 investigated MM-302 (30 mg/m^2^) on day 1 cycle 1, initially in three patients; in subsequent cycles patients received trastuzumab (6* or 8 mg/kg [*with 8 mg/kg loading] every 3 weeks [q3w]) on day 1 followed by MM-302 on day 6. After safety had been established in the first cohort, a larger expansion cohort (cohort 2) of nine patients investigated trastuzumab administered on cycle 1 day 1, followed by MM-302 and ^64^Cu-MM-302 on cycle 1 day 6. During subsequent cycles, trastuzumab was administered q3w on day 1 and a full therapeutic dose of non-radioactive MM-302 (30 mg/m^2^) was administered q3w on day 6. Similarly, arm 4 investigated intravenous cyclophosphamide (450 mg/m^2^ q3w) and trastuzumab (6 mg/kg after 8 mg/kg loading) on day 1 followed by MM-302 (30 mg/m^2^ q3w) on day 6 in 13 patients. Cyclophosphamide administration in arm 4 continued beyond cycle 4 at physician discretion and depending on tolerability.

**Table 1:** Treatment administration and dose-limiting toxicity

	Treatment administered			*N*	Median (range) time on treatment, months	Dose-limiting toxicity, *n*	Details of dose-limiting toxicity
Arm 1: MM-302 monotherapy							
Cohort	MM-302 dose, mg/m^2^, q4w						
1	8			3	3·2 (1·3– 3·7)	0	
2	16			4	1·3 (0·5–2·4)	0	
3	30			3	1·9 (1·6–11·1)	0	
4	40			12	8·0 (1·0–48·5)	0	
5	50			12	2·6 (1·4–8·9)	0	
Arm 2: MM-302 + trastuzumab							
Cohort	MM-302 dose, mg/m^2^, q4w	Trastuzumab dose, mg/kg, q2w^a^					
1	30	4		6	4·2 (1·8–19·3)	1	Febrile neutropenia
2	40	4		4	7·2 (1·9–12·7)	0	
Arm 3: MM-302 + trastuzumab							
Cohort	MM-302 dose, mg/m^2^, q3w^c^	Trastuzumab dose, mg/kg, q3w^b^					
1	30	6^d^		3	2·1 (1·4–8·1)	0	
2	30	6		9	1·7 (0·5–8·3)	0	
Arm 4: MM-302 + trastuzumab + cyclophosphamide							
Cohort	MM-302 dose, mg/m^2^, q3w^c^	Trastuzumab dose, mg/kg, q3w^b^	Cyclophosphamide dose, mg/m^2^, q3w^e^				
1	30	6	600	13	3·0 (0·7–28·8)	0	

^a^The first dose was a 6 mg/kg loading dose

^b^The first dose was an 8 mg/kg loading dose

^c^For the first cycle, ~3–5 mg/m^2 64^Cu-MM-302 was administered for the first cycle only in addition to the MM-302 dose

^d^In arm 3, cohort 1, trastuzumab was administered from cycle 2 onwards

^e^Cyclophosphamide continued beyond cycle 4 at physician discretion and depending on tolerability

Following treatment with unlabeled MM-302, patients in arms 3 and 4 also received 337–432 MBq ^64^Cu-MM-302 (^64^Cu-MM-302), mass dosage 3–5 mg/m^2^, during cycle 1, on the first day of MM-302 administration only, and then 2–3 positron emission tomography/computed tomography (PET/CT) scans were obtained immediately after administration and up to 2 days later to assess ^64^Cu-MM-302 biodistribution and tumor deposition.^23,24^

Patients were treated until disease progression, unacceptable toxicity, or withdrawal of consent. A 25% dose reduction was permitted for any toxicity ≥ grade 3 that was considered related and/or significant by the investigator. Treatment could be paused for ≤56 days to allow recovery from an adverse event (AE); otherwise treatment was discontinued.

Patients experiencing an asymptomatic reduction in LVEF to <50% or >10% absolute drop from baseline could continue treatment at the discretion of the medical monitor, provided that cardiac function tests were performed prior to each MM-302 dose. Study treatment was discontinued permanently if LVEF did not improve or declined further.

From September 2012, all patients starting treatment received prophylactic premedication with 25–50 mg diphenhydramine or equivalent (administered orally ≤30 min prior to MM-302 dosing or intravenously immediately prior to MM-302 dosing) in cycle 1. Patients who tolerated the initial dose without an infusion reaction could discontinue premedication in subsequent cycles according to investigator judgment.

### Assessments

DLTs were evaluated during cycle 1 of treatment and AEs were monitored throughout treatment and for 30 days after administration of the last study drug dose, according to the National Cancer Institute Common Terminology Criteria for Adverse Events (NCI CTCAE) version 4·0.

Cardiac function was regularly monitored throughout the study. Electrocardiograms were obtained at screening, prior to each cycle, at study end, and at 30-day follow-up. Radionuclide ventriculography or echocardiography was obtained at screening, every 4 weeks for the first three cycles then every 8 weeks thereafter, at study end, and at 30-day follow-up.

Blood samples for PK analysis were collected immediately before and after MM-302 infusion, and at approximately 2, 4, 8, 24, 48, and 72 h after the start of infusion, 8 days after treatment, at study termination, and at 30-day follow-up. Samples were analysed for total and encapsulated doxorubicin, as well as the anti-HER2 single chain variable fragment (scFv), F5. Analysis was performed comparing PK parameters to drug activity. Anti-drug antibody samples were collected before dosing and at the onset of any infusion reaction, at the resolution of the event, and at 28 days after the event.

ORR was assessed by RECIST version 1·1 at screening and every 8 weeks from first dose. CBR, median duration of response, and median PFS were calculated per dose cohort and for the overall population.

Tumor biopsy samples were collected 72 h after MM-302 dosing to measure HER2, microvessel density, and microscopic distribution of MM-302 within the tumor. A subgroup of patients received ^64^Cu-MM-302 to map biodistribution and tumor deposition, under a companion imaging protocol described elsewhere.^23,24^

### Statistical analysis

Patients who received at least a partial infusion of MM-302 during this period were included in the safety and efficacy population. Efficacy was also reported separately for patients who received ≥30 mg/m^2^ MM-302. Safety, efficacy, and PK parameters were summarized using descriptive statistics for continuous variables and frequency distributions for categorical variables.

## Results

Seventy-three patients were enrolled between 25 July 2011 and 24 June 2014, 69 of whom received study treatment in four arms with MM-302 either alone or in combination with trastuzumab and cyclophosphamide (Table 1 and Supplemental Fig. 2). Patient demographics and characteristics are described in Table 2. At data cutoff (18 August 2016), study treatment was ongoing for one patient receiving MM-302 monotherapy and one patient receiving MM-302 plus trastuzumab and cyclophosphamide (Supplemental Fig. 2).

**Table 2:** Patient baseline demographics

	Arm 1	Arm 2	Arm 3	Arm 4	Total (safety population)
Female gender, *n* (%)	34 (100)	10 (100)	12 (100)	13 (100)	69 (100)
Median (range) age	55·0 (31–75)	53·5 (45–68)	48·5 (37–65)	58·0 (43–71)	55·0 (31–75)
Ethnicity, *n* (%)					
Caucasian	32 (94)	9 (90)	10 (83)	13 (100)	64 (93)
Black	2 (6)	0	2 (17)	0	4 (6)
Asian	0	1 (10)	0	0	1 (1)
ECOG performance status, *n* (%)					
0	16 (47)	5 (50)	7 (58)	5 (39)	33 (48)
1	18 (53)	5 (50)	5 (42)	6 (62)	36 (52)
Median (range) time from first diagnosis, months	72·8 (7·3–311·0)	89·7 (8·1–144·5)	46·3 (10·8–199·6)	85·4 (11·0–198·6)	69·1 (7·3–311·0)
Median (range) time from first diagnosis of metastatic disease, months	33·6 (0·1–199·5)	47·8 (5·0–101·1)	29·0 (7·6–76·1)	54·45 (1·2–198·0)	39·5 (0·1–199·5)
Disease status, *n* (%)					
Locally advanced	0	0	0	2 (15)	2 (3)
Distant metastases	34 (100)	10 (100)	12 (100)	11 (85)	67 (97)
Disease stage at diagnosis, *n* (%)					
IA	5 (15)	1 (10)	0	3 (23)	9 (13)
IB	1 (3)	0	0	0	1 (1)
IIA	6 (18)	2 (20)	1 (8)	2 (15)	11 (16)
IIB	5 (15)	2 (20)	1 (8)	1 (8)	9 (13)
IIIA	4 (12)	2 (20)	1 (8)	0	7 (10)
IIIB	0	0	0	1 (8)	1 (1)
IIIC	0	0	1 (8)	0	1 (1)
IV	4 (12)	1 (10)	6 (50)	3 (23)	14 (20)
Unknown	9 (27)	2 (20)	2 (17)	3 (23)	16 (23)
ER receptor status, *n* (%)					
Positive	6 (18)	4 (40)	1 (8)	2 (15)	13 (19)
Negative	1 (3)	2 (20)	6 (50)	3 (23)	12 (17)
Unknown	27 (79)	4 (40)	5 (42)	8 (62)	44 (64)
PR receptor status, *n* (%)					
Positive	3 (9)	2 (20)	2 (16)	1 (8)	8 (11)
Negative	4 (12)	4 (40)	5 (42)	4 (31)	17 (25)
Unknown	27 (79)	4 (40)	5 (42)	8 (61)	44 (64)
Median (range) number of prior anticancer therapies	5 (1–10)	4 (1–12)	4·5 (2–10)	5 (2–10)	5 (1–12)
Previous exposure to therapy, *n* (%)					
Trastuzumab	34 (100)	9 (90)	12 (100)	13 (100)	68 (99)
Taxane	33 (97)	9 (90)	11 (92)	11 (85)	64 (93)
Lapatinib	23 (68)	6 (60)	6 (50)	7 (54)	42 (61)
Anthracycline	18 (53)	6 (60)	5 (42)	8 (62)	37 (54)
Ado-trastuzumab emtansine	10 (29)	4 (40)	11 (92)	10 (77)	35 (51)
(T-DM1)					
Hormonal therapy	18 (53)	4 (40)	3 (25)	8 (62)	33 (48)
Pertuzumab	1 (3)	5 (50)	8 (67)	3 (23)	17 (25)
Prior surgery, *n* (%)	33 (97)	9 (90)	11 (92)	12 (92)	65 (94)
Prior radiotherapy, *n* (%)	23 (68)	7 (70)	9 (75)	8 (62)	47 (68)

*CISH* chromogenic in situ hybridization, *ECOG* Eastern Cooperative Oncology Group, *ER* estrogen receptor, *FISH* fluorescence in situ hybridization, *HER2* human epidermal growth factor receptor 2, *IHC* immunohistochemistry, *PR* progesterone receptor

Patients remained on study treatment for a median of 3·0 months (range: 0·5–48·5). Of note, as of data cutoff, one patient had received >49 cycles of 40 mg/m^2^ MM-302 q4w for a cumulative dose of 1960 mg/m^2^ MM-302 and was continuing to receive study treatment.

The most common AEs of any grade were fatigue and nausea (MM-302 monotherapy plus trastuzumab), or nausea and vomiting (MM-302 plus trastuzumab and cyclophosphamide) (Table 3). Specific AEs for doxorubicin, e.g., cardiac toxicity, were followed as AEs of special interest (Supplemental Table 1). Grade 3 or 4 treatment-emergent AEs occurred in 24/69 patients (Table 3); neutropenia was the most common in 6/34 (18%) patients receiving monotherapy and 1/13 (8%) patients receiving MM-302 plus trastuzumab. Median time to absolute neutrophil count nadir was 23 days. The highest incidence of grade 3/4 AEs was seen with 50 mg/m^2^ MM-302 monotherapy (*n* = 5/12; 42%). As this occurred beyond cycle 1, neutropenia did not meet the criteria of DLT. Neutropenia was primarily observed in patients receiving higher dose intensities (>10 mg/m^2^/week). In addition, grade 3/4 neutropenia was observed only in patients previously treated with an anthracycline (Supplemental Figure 4).

**Table 3:** Most common treatment-emergent adverse events all grade occurring in >20% of patients in the overall population and grade 3/4 adverse events occurring in more than two patients in any treatment arm (safety population)

	Arm 1(*n* = 34)		Arm 2 (*n* = 10)		Arm 3 (*n* = 12)		Arm 4 (*n* = 13)		Total (*n* = 69)	
	All AEs	G3/4	All AEs	G3/4	All AEs	G3/4	All AEs	G3/4	All AEs	G3/4
Fatigue	21 (62)	0	4 (40)	0	6 (50)	1 (11)	3 (23)	1 (8)	34 (49)	2 (3)
Nausea	19 (56)	0	2 (20)	0	7 (58)	0	6 (46)	0	34 (49)	0
Decreased appetite	13 (38)	0	2 (20)	0	3 (25)	0	2 (15)	0	20 (29)	0
Vomiting	9 (27)	0	1 (10)	0	4 (33)	0	5 (39)	0	19 (28)	0
Cough	8 (24)	0	2 (20)	0	5 (42)	0	3 (23)	0	18 (26)	0
Diarrhea	7 (21)	0	3 (30)	0	3 (25)	0	4 (31)	0	17 (25)	0
Constipation	7 (21)	0	2 (20)	0	3 (25)	0	4 (31)	0	16 (23)	0
Stomatitis	8 (24)	0	4 (40)	0	2 (17)	0	2 (15)	0	16 (23)	0
Dyspnea	5 (15)	2 (6)	2 (20)	0	5 (42)	0	4 (31)	0	16 (23)	0
Neutropenia	9 (27)	6 (18)	0	0	3 (25)	0	3 (23)	1 (8)	15 (22)	7 (10)
Rash	9 (27)	0	1 (10)	0	1 (8)	0	3 (23)	0	14 (20)	0
Infusion-related reaction	5 (15)	0	2 (20)	0	5 (42)	0	2 (15)	0	14 (20)	0
Mucosal inflammation	5 (15)	2 (6)	1 (10)	0	4 (33)	0	3 (23)	0	13 (19)	2 (3)
Leukopenia	5 (15)	2 (6)	0	0	0	0	1 (8)	0	6 (9)	2 (3)

The additional following grade 3/4 adverse events were each seen in one patient only in the separate treatment arms: anemia, lymphopenia, thrombocytopenia, alanine aminotransferase increased, aspartate aminotransferase increased, bilirubin conjugated increased, hematocrit increased, depression, palmar-plantar erythrodysesthesia syndrome, (arm 1); febrile neutropenia, neutropenic infection, lymphoedema, (arm 2); influenza, presyncope, hip fracture, blood alkaline phosphatase increased, blood bilirubin increased, hyponatremia, cognitive disorder, mental status changes, breast pain, pulmonary embolism, (arm 3); lymphopenia, white blood cell count decreased, hypoalbuminemia, hyponatremia (arm 4)

Seventeen patients had ≥1 dose interruption, and of these, 15 patients had ≥1 dose interruption due to infusion reaction. Eight patients discontinued treatment due to an AE (*n* = 1 for each of convulsion; aphasia/face edema; peripheral sensory neuropathy; cardiac failure [see below]; neutropenia; hypoxia; thrombocytopenia; back pain/dyspnea/infusion-related reaction). Three patients experienced ≥1 treatment-related serious AE (anemia/neutropenia/thrombocytopenia; palmar-plantar erythrodysesthesia syndrome; febrile neutropenia). Only one DLT (febrile neutropenia) was observed in a patient treated with 30 mg/m^2^ MM-302 q4w plus 4 mg/kg trastuzumab q2w (Table 1). There were no treatment-related deaths. The MTD was not defined in this trial; however, post-cycle 1 neutropenia prompted the recommendation of a phase 2 dose of 30 mg/m^2^ q3w or 40 mg/m^2^ q4w.

**Fig. 1:**
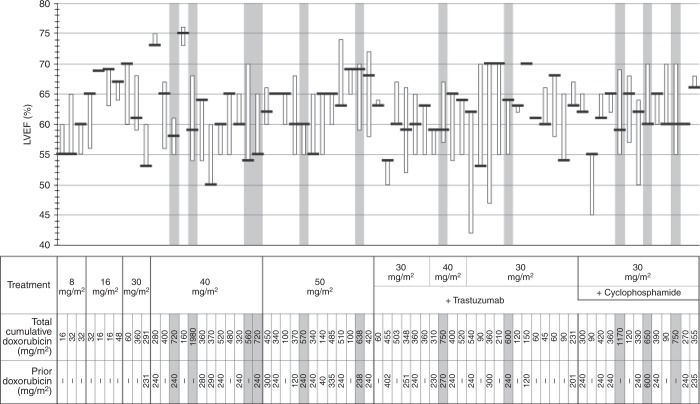
Cardiac safety. Left ventricular ejection fraction (LVEF) is shown by patient (black horizontal line denotes baseline value, box denotes range over treatment period). Treatment received, including dose of MM-302, is shown below graph for each patient as well as cumulative doxorubicin exposure. Grey highlighting shows the 11 patients with cumulative anthracycline exposure >550 mg/m^2^

There were no reported cardiac AEs ≥grade 2 with MM-302 monotherapy, and cardiac AEs were infrequent in the MM-302 combination arms with no serious AEs. Six patients on the combination arms had a protocol-defined asymptomatic decline in LVEF defined as (1) a post-baseline measurement <50%, or 2) a post-baseline measurement that was a reduction of > 10 absolute percentage points from baseline (Fig. 1): three patients had LVEF < 50%; four patients had reversible declines; one patient had a decline to 47% noted at off-study assessment; one patient (receiving 30 mg/m^2^ MM-302 and 6 mg/kg trastuzumab q3w) was observed twice to have a range of 45–50% LVEF (unchanged off-study). This patient discontinued treatment because of LVEF decline. Eleven patients had lifetime cumulative anthracycline exposure >550 mg/m^2^ with no protocol-defined LVEF changes (Fig. 1).

Total doxorubicin, encapsulated doxorubicin, and anti-HER scFv F5 plasma concentrations were measured (Supplemental Fig. 3) and pharmacokinetic parameters summarized in Supplemental Table 2. The terminal half-lives at the first cycle are estimated using non-compartmental analysis to be 47·3, 44·8, and 63·3 h for total doxorubicin, encapsulated doxorubicin, and F5, and these values increased by ~15% in subsequent cycles. Measurements of total and encapsulated doxorubicin indicated that the two analytes are highly correlated; 98% doxorubicin remained encapsulated in samples collected up to 1 week. Area under the curve (AUC) and maximum concentration (*C*_max_) of total and encapsulated doxorubicin and F5 increased proportionally with dose.

In four patients, positively confirmed anti-drug antibodies (ADAs) non-specific to MM-302 were detected at baseline, prior to any MM-302 treatment, and were also detected post-baseline. In two other patients, ADAs were detected post-baseline at cycle 2 and 6 and titer levels remained ≤ 40 relative luminescence units (RLU); both had negative ADAs at study end.

The patient population in this trial was heavily pretreated, with a median of five prior therapies and 99% of patients had received prior trastuzumab. Overall, ORR was 12% (*n* = 8/69; 95% CI: 5·1, 21·6) and CBR was 28% (*n* = 19/69; 95% CI: 17·5, 39·6). No responses were observed in patients receiving <30 mg/m^2^ of MM-302. Median PFS was 4·4 months (95% CI: 3·1, 8·6).

**Fig. 2:**
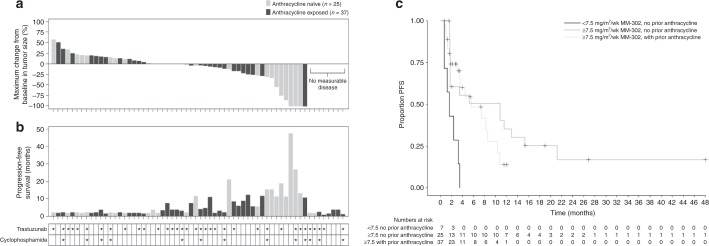
**a** Maximum change from baseline in tumor size (sum of target lesions) and progression-free survival (PFS). **b** Maximum change from baseline in tumor size (sum of target lesions) and PFS in patients treated with MM-302 ≥30 mg/m^2^ (safety population). **c** Kaplan-Meier graph of PFS in subgroups split by average MM-302 dose intensity and prior anthracycline status (<7.5 mg/m^2^/week, ≥7.5 mg/m^2^/week with and without prior anthracycline)

In patients receiving ≥30 mg/m^2^ MM-302 (*n* = 62: 27 monotherapy; 22 plus trastuzumab; 13 plus trastuzumab/cyclophosphamide), ORR was 13% (*n* = 8/62; two CR and six PR) (Fig. 2a), CBR was 30·6% (*n* = 19/62) and median PFS was 7·4 months (95% CI: 3·5–10·9) (Fig. 2b). Among patients receiving ≥30 mg/m^2^ MM-302, in the anthracycline-exposed group (*n* = 37), ORR was 3% (*n* = 1/37), CBR was 24% (*n* = 9/37) and median PFS 5·6 months (95% CI: 3·5–8·6). In the anthracycline-naïve subgroup (*n* = 25), ORR was 28% (*n* = 7/25), CBR was 40% (*n* = 10/25) and median PFS was 10·9 months (95% CI: 1·8−15·3) (Fig. 2a, b). A relationship was observed between clinical efficacy and dose, suggesting that a greater reduction in tumor burden was observed at doses ≥30 mg/m^2^ (Fig. 2c and Supplementary Figure 5).

**Fig. 3:**
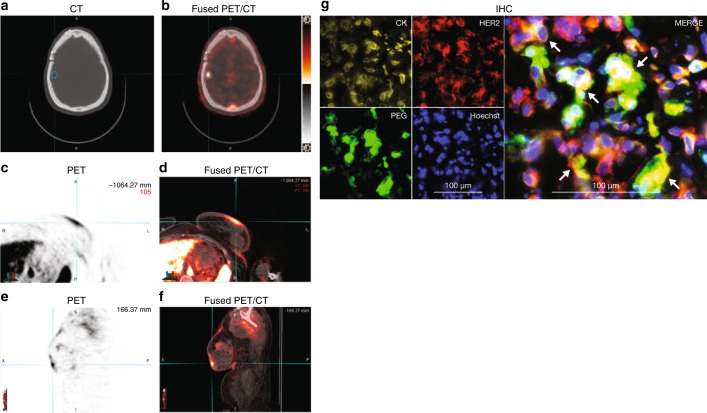
**a, b** Are representative axial CT and PET images in two patients with HER2-positive metastatic breast cancer at 24-h post-administration of ^64^Cu-MM-302, respectively, of the head. Brain lesion (as confirmed by MRI at screening) is indicated by the blue contour and is shown to have significant uptake of ^64^Cu-MM-302 as seen in (**b**). **c–f** Show ^64^Cu-MM-302 deposition in a skin lesion in another patient. **c**–**f** Are representative axial and sagittal views, respectively, of the skin lesion (as indicated by the cyan cross hair marker). **g** Immunofluorescence images (20x) of a day 3 liver biopsy of a study patient (IHC 3+ and FISH positive on FFPE) treated with 30 mg/m^2^ MM-302 q4w. The biopsy was stained for cytokeratin (CK, yellow) to identify tumor cells, HER2 (red), PEG (green) to identify MM-302, and nuclei with Hoechst (blue) (left four panels). Merged images (right panel) show clusters of HER2-positive tumor cells with internalized liposomes (arrows). CT computed tomography, HER2 human epidermal growth factor receptor 2, MRI magnetic resonance imaging, PET positron emission tomography, FFPE formalin-fixed paraffin-embedded, FISH fluorescence in situ hybridization, HER2 human epidermal growth factor receptor 2, IHC immunohistochemistry, PEG polyethylene glycol, q4w every 4 weeks

PET/CT following administration of ^64^Cu-MM-302 revealed diverse locations of tumor MM-302 uptake in different patients, including skin, breast, liver, and brain (Fig. 3a–f), and with different lesion intensities both intra- and interpatient.^23^ High levels of MM-302 in the blood and liver at the time points used for imaging were consistent with the anticipated biodistribution for a PEGylated liposomal agent. Furthermore, HER2-mediated delivery of MM-302 to HER2-overexpressing tumor cells was confirmed in multiple evaluable biopsies post-MM-302 treatment by co-localization of anti-PEG, anti-HER2, and anti-cytokeratin antibodies (Fig. 3g).

## Discussion

In this phase 1, open-label, dose-escalation study, MM-302 was well tolerated and active in patients with HER2-positive advanced/metastatic breast cancer. The study population was heavily pretreated with a median of five prior regimens for metastatic disease. The main study objective was to determine the safety of MM-302 as a monotherapy and in combination with trastuzumab and cyclophosphamide. Consistent with the nature of a phase 1 dose escalation study, conclusions on efficacy are confounded by the heterogeneous doses and schedules of MM-302 used and the multitude of monotherapy and combination therapy cohorts. Nonetheless, there is notable lasting activity without cumulative toxicity.

MM-302 was designed to target delivery of doxorubicin to tumors via anti-HER2-directed antibodies. Owing to the low level of HER2-expression on cardiomyocytes, preclinical studies with MM-302 show no uptake into human stem cell-derived cardiomyocytes and no effect on cell viability.^18^ However, to safeguard against cardiac toxicity, effects on the heart were monitored very closely. No cardiac AEs were observed in patients treated with MM-302 monotherapy. With MM-302 in combination with trastuzumab, with or without cyclophosphamide, cardiac AEs were infrequent with no serious AEs.

MM-302 activity correlated with AUC and, in general, higher activity was observed at doses ≥10 mg/m^2^/week (30 mg/m^2^ q3w and 40 mg/m^2^ q4w) (Fig. 3 and Supplemental Figure 5). Although MTD was not reached at 50 mg/m^2^ q4w (12·5 mg/m^2^/week), dose intensity of 10 mg/m^2^/week was also active and had fewer cumulative incidences of neutropenia. Based on phase 1 results, the recommended phase 2 dose was 30 mg/m^2^ q3w MM-302 (10 mg/m^2^/week) in combination with trastuzumab 6 mg/kg q3w (with 8 mg/kg loading).

The observed activity of MM-302 is promising. Median PFS of 7·4 months with ≥30 mg/m^2^ MM-302 monotherapy, in combination with trastuzumab or with trastuzumab and cyclophosphamide, compares favourably with recent late-stage HER2-positive clinical trials that demonstrated median PFS of 3·3 months in patients treated with the physician’s choice.^25^ Furthermore, in the present study, the observed PFS and ORR in patients not previously exposed to an anthracycline were 10·9 months and 28%, respectively. Multiple studies of liposomal doxorubicin have observed similar trends, with improved activity in patients not previously exposed to an anthracycline compared to those previously exposed.^12,20,21^ Additionally, grade 3 palmar-plantar erythrodysesthesia with MM-302 (*n* = 1/69; 1%) was less frequent than that reported for the combination of liposomal doxorubicin plus trastuzumab (*n* = 9/30; 30%; respectively).^9^

Exploratory analyses of tumor biopsies support a HER2-targeting mechanism for MM-302 consistent with preclinical data. Tumor deposition data using ^64^Cu-MM-302 PET/CT revealed the potential of MM-302 to be delivered to difficult metastatic locations such as brain metastases.^23^

In summary, these data suggest that MM­302 monotherapy, in combination with trastuzumab or trastuzumab and cyclophosphamide has a manageable safety profile and promising clinical activity in patients with advanced HER2-positive breast cancer. MM-302 30 mg/m^2^ in combination with 6 mg/kg trastuzumab every 3 weeks was selected as the recommended phase 2 study dose. This study is now completed, and further clinical studies are under discussion.

## Electronic supplementary material

Supplementary Information_Revised_Clean
