# Supplementary material for: Safety and pharmacokinetics of MM-302, a HER2-targeted antibody–liposomal doxorubicin conjugate, in patients with advanced HER2-positive breast cancer: a phase 1 dose-escalation study
Source: Br J Cancer. 2018 Oct 26;119(9):1086–93. doi: 10.1038/s41416-018-0235-2 (PMC6219487; doi:10.1038/s41416-018-0235-2)

# Supplementary Information

## Dose-limiting toxicities

Dose-limiting toxicities were evaluated during treatment cycle 1 and defined as:

Grade 3 or higher febrile neutropenia or ≥grade 3 neutropenia with infection or grade 4 neutropenia lasting more than 7 days.

Grade 4 thrombocytopenia (or grade 3 with significant bleeding).

Grade 3 or 4 treatment-related non-hematological toxicity, except nausea, vomiting, diarrhea, and elevations in aspartate aminotransferase (AST) which will be considered dose limiting only if the patient develops:

- - Grade 3 or higher nausea, vomiting, or diarrhea lasting more than 48 hours despite use of appropriate therapy.
  - Grade 3 elevations in AST lasting longer than one week, or grade 4 elevation in AST.
  - Alkaline phosphatase (ALP) elevations will be evaluated on a case by case basis by the study team in assessing whether the event is a dose-limiting toxicity.
  - Grade 3 hypersensitivity reaction lasting longer than 24 hours or that does not allow for the entire dose of the drug to be administered, or grade 4 hypersensitivity regardless of duration.

Dosing delay >1 cycle due to a study drug related adverse event or related severe laboratory abnormalities.

Supplemental Table 1. Treatment-emergent adverse events (all grades) of special interest (safety population)

|  | Arm 1 (n=34) | Arm 2 (n=10) | Arm 3 (n=12) | Arm 4 (n=13) | Total (n=69) |
| --- | --- | --- | --- | --- | --- |
| Mucositis^1^ | 12 (35) | 5 (50) | 6 (50) | 4 (31) | 27 (39) |
| Neutropenia | 9 (26) | 0 | 3 (25) | 3 (23) | 15 (22) |
| Infusion reaction | 5 (15) | 2 (20) | 5 (42) | 2 (15) | 14 (20) |
| Anemia^2^ | 3 (9) | 2 (20) | 1 (8) | 3 (23) | 9 (13) |
| Thrombocytopenia | 4 (12) | 0 | 0 | 4 (31) | 8 (12) |
| Alopecia | 4 (12) | 1 (10) | 1 (8) | 2 (15) | 8 (12) |
| Leukopenia^3^ | 6 (18) | 0 | 0 | 2 (15) | 8 (12) |
| Palmar-plantar erythrodysesthesia^4^ | 3 (9) | 1 (10) | 0 | 0 | 4 (6) |
| Cardiac failure | 0 | 0 | 1 (8) | 0 | 1 (1) |
| Neutropenic fever | 0 | 1 (10) | 0 | 0 | 1 (1) |

^1^Mucositis consists of MedDRA preferred terms stomatitis and mucosal inflammation.

^2^Anemia consists of MedDRA preferred terms anemia, hematocrit decreased, and red blood cell count decreased.

^3^Leukopenia consists of leukopenia and white blood cell count decreased.

^4^Palmar-plantar erythrodysesthesia consists of palmar erythema and palmar-plantar erythrodysesthesia syndrome.

Supplemental Table 2. Summary of estimated pharmacokinetic parameters for total doxorubicin, encapsulated doxorubicin, and anti-HER2 scFv, F5

| Analyte | Parameter | Cycle | n | Geometric mean | 95% CI | |
| --- | --- | --- | --- | --- | --- | --- |
| Total doxorubicin | Clearance (L/week) | Cycle 1 | 39 | 4·53 | 3·90 | 5·27 |
|  |  | Cycle 2+ | 57 | 4·13 | 3·64 | 4·68 |
|  | Volume (L/m^2^) | Cycle 1 | 39 | 1·84 | 1·75 | 1·94 |
|  |  | Cycle 2+ | 57 | 1·77 | 1·67 | 1·89 |
|  | Half-life (hours) | Cycle 1 | 39 | 47·3 | 41·9 | 53·4 |
|  |  | Cycle 2+ | 57 | 50·0 | 45·4 | 55·1 |
| Encapsulated doxorubicin | Clearance (L/week) | Cycle 1 | 39 | 4·63 | 3·99 | 5·37 |
|  |  | Cycle 2+ | 57 | 4·18 | 3·59 | 4·88 |
|  | Volume (L/m^2^) | Cycle 1 | 39 | 1·78 | 1·68 | 1·90 |
|  |  | Cycle 2+ | 57 | 1·78 | 1·65 | 1·93 |
|  | Half-life (hours) | Cycle 1 | 39 | 44·8 | 39·1 | 51·3 |
|  |  | Cycle 2+ | 57 | 49·7 | 44·3 | 55·7 |

| scFv F5 | Clearance (L/week  ng F5/mg total doxorubicin) | Cycle 1 | 34 | 0·11 | 0·09 | 0·15 |
| --- | --- | --- | --- | --- | --- | --- |
|  |  | Cycle 2+ | 52 | 0·11 | 0·09 | 0·14 |
|  | Volume (L/m^2^  ng F5/mg total doxorubicin) | Cycle 1 | 34 | 0·06 | 0·06 | 0·07 |
|  |  | Cycle 2+ | 52 | 0·06 | 0·06 | 0·07 |
|  | Half-life (hours) | Cycle 1 | 34 | 63·3 | 49·4 | 81·1 |
|  |  | Cycle 2+ | 52 | 67·7 | 54·9 | 83·5 |

CI=confidence interval; HER2=human epidermal growth factor receptor 2; scFv=single-chain variable fragment.

# Supplemental figures

Supplemental Figure 1. MM-302 structure and mechanism of action. These diagrams show MM-302 structure (A); MM-302 and trastuzumab binding to the HER2 receptor (B); and targeted delivery of MM-302 to HER-overexpressing tumor cells while sparing normal cardiomyocytes (C). DSPE=distearoylphosphatidylethanolamine; HER2=human epidermal growth factor receptor 2; PEG=polyethylene glycol; scFv=single chain variable fragment. Reproduced from Miller K et al. BMC Cancer 2016;16:352–62, under Creative Commons Attribution 4.0


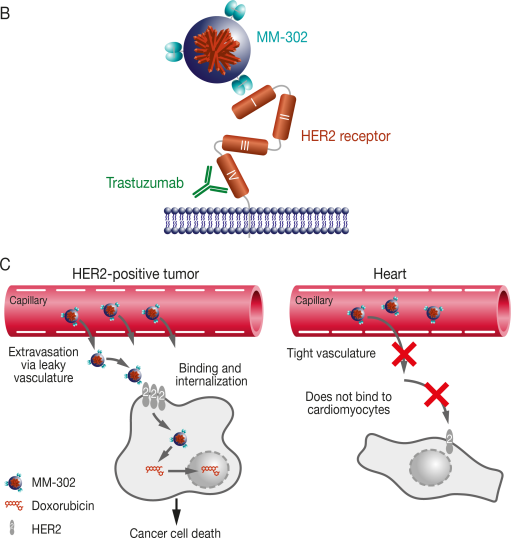

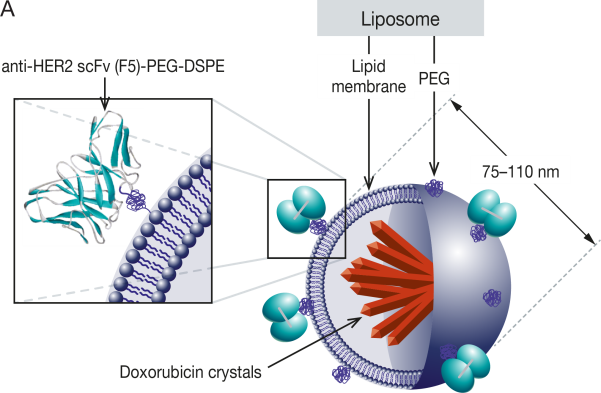
International License (<http://creativecommons.org/licenses/by/4.0/>).

Supplemental Figure 2. Consort diagram. q3w=every 3 weeks; q4w=every 4 weeks ^a^6 mg/kg trastuzumab loading dose, ^b^8 mg/kg trastuzumab loading dose.


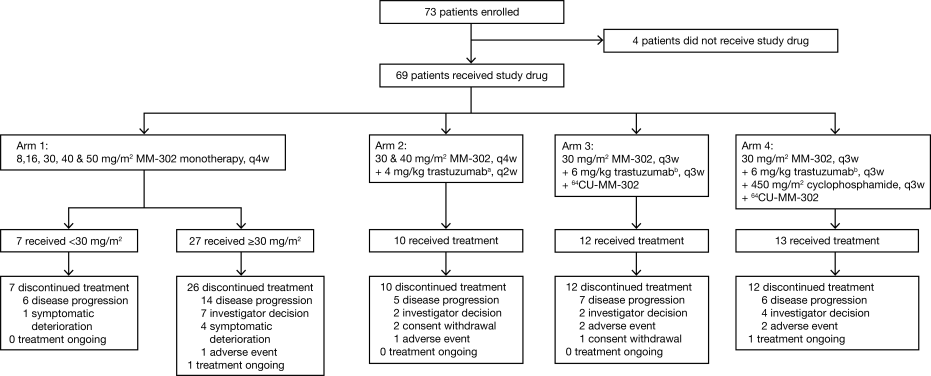


Supplemental Figure 3. Pharmacokinetics of MM-302, as assessed by total doxorubicin, are shown for dose regimens indicated. q3w=every 3 weeks; q4w=every 4 weeks.


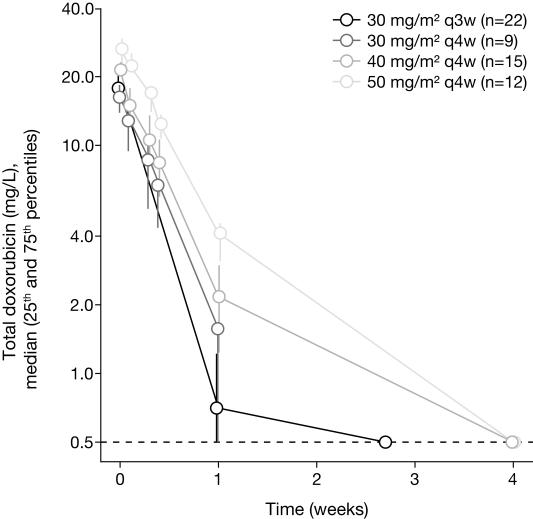


Supplemental Figure 4. Probability of neutropenia incidence (upper panels, any grade; lower panels, grade 3/4) as a function of MM-302 dose intensity and prior anthracycline (left panels, no prior anthracycline; right panels, with prior anthracycline)


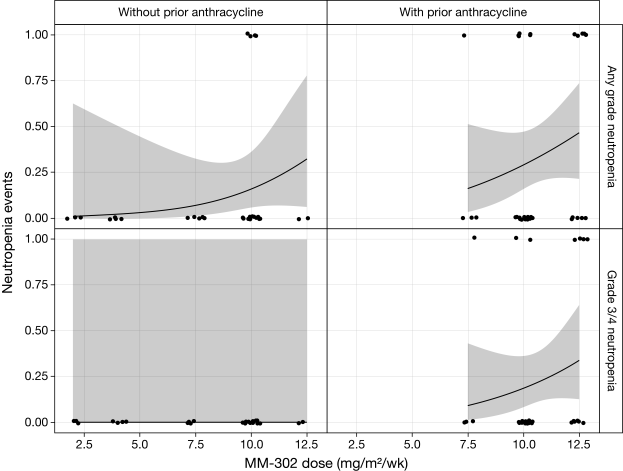


Supplemental Figure 5. Probability of having a CR or PR as a function of MM-302 dose intensity for patients without (left panel) and with (right panel) prior anthracycline exposure


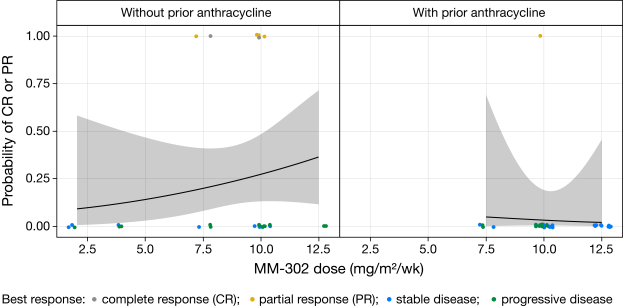

Supplement: Supplementary file 1 — Supplementary Information_Revised_Clean [file 41416_2018_235_MOESM1_ESM.docx]
